# Alterations in Lipid Mediated Signaling and Wnt/**β**-Catenin Signaling in DMH Induced Colon Cancer on Supplementation of Fish Oil

**DOI:** 10.1155/2014/832025

**Published:** 2014-06-05

**Authors:** Shevali Kansal, Kim Vaiphei, Navneet Agnihotri

**Affiliations:** ^1^Department of Biochemistry, Panjab University, Chandigarh 160014, India; ^2^Department of Histopathology, Post Graduate Institute of Medical Education and Research (PGIMER), Chandigarh 160012, India

## Abstract

Ceramide mediates inhibition of cyclooxygenase-2 (COX-2) which catalyzes formation of prostaglandin further activating peroxisome proliferator-activated receptor**γ** (PPAR**γ**) and Wnt/**β**-catenin pathway; and hence plays a critical role in cancer. Therefore, in current study, ceramide, COX-2, 15-deoxy prostaglandin J_2_(15-deoxy PGJ_2_), PPAR**γ**, and **β**-catenin were estimated to evaluate the effect of fish oil on lipid mediated and Wnt/**β**-catenin signaling in colon carcinoma. Male Wistar rats in Group I received purified diet while Groups II and III received modified diet supplemented with FO : CO(1 : 1) and FO : CO(2.5 : 1), respectively. These were further subdivided into controls receiving ethylenediaminetetraacetic acid and treated groups receiving dimethylhydrazine dihydrochloride (DMH)/week for 4 weeks. Animals sacrificed 48 hours after last injection constituted initiation phase and those sacrificed after 16 weeks constituted postinitiation phase. Decreased ceramide and increased PPAR**γ** were observed in postinitiation phase only. On receiving FO+CO(1 : 1)+DMH and FO+CO(2.5 : 1)+DMH in both phases, ceramide was augmented whereas COX-2, 15-deoxy PGJ_2_, and nuclear translocation of **β**-catenin were reduced with respect to cancerous animals. Decrease was more significant in postinitiation phase with FO+CO(2.5 : 1)+DMH. Treatment with oils increased PPAR**γ** in initiation phase but decreased it in postinitiation phase. Hence, fish oil altered lipid mediated signalling in a dose and time dependent manner so as to inhibit progression of colon cancer.

## 1. Introduction

Recent reports have shown that lipid mediated signaling triggers a systemic physiological response which results in the pathogenesis of cancer [1, 2]. The sphingolipids have emerged as important lipid signaling molecules regulating fundamental cell responses such as cell death and differentiation, proliferation, and aspects of inflammation. Two major sphingolipid metabolites involved in signaling pathways are ceramide and sphingosine-1-phosphate (S1P). Ceramide is intimately involved in a myriad of physiological processes including cell growth or death, cell adhesion, cell migration, inflammation, and angiogenesis [3]. It acts as a second messenger for multiple extracellular stimuli including growth factors and environmental stresses, such as hypoxia, heat stress, and irradiation [4]. It transduces the signaling pathwaysby regulating specific protein targets such as phosphatases and kinases [5]. These protein targets, in turn, modulate the components of signalling pathways, for example, Akt, phospholipase D, protein kinase C (PKC), c-Raf, and kinase suppressor of Ras [6]. Therefore, defects in ceramide generation lead to the survival of cancer cell which further develops into tumor [4]. Another important mediator of lipid signaling is the enzyme cyclooxygenase-2 (COX-2). It is an inducible enzyme and is involved in the modulation of inflammation by catalyzing the rate-limiting step that leads to the formation of prostaglandins (PGs) from arachidonic acid. Studies have shown that cyclooxygenase-2 (COX-2) is overexpressed in various cancers and leads to the enhancement in production of inflammatory prostaglandins such as 15-deoxy delta (12,14) prostaglandin J_2_ (15-deoxy PGJ_2_) [7, 8]. 15-deoxy PGJ_2_, being a natural ligand, activates peroxisome proliferator-activated receptor *γ* (PPAR*γ*) which then forms the heterodimer with retinoid X receptor [9, 10]. This complex binds to a specific DNA sequence [PPAR response element (PPRE)] and stimulates transcription of genes involved in cell growth and apoptosis [11]. The inflammatory prostanoids also increased the release of glycogen synthase kinase 3*β* (GSK3*β*) from axin complex, thereby relieving *β*-catenin and leading to the activation of Wnt signaling pathway. *β*-Catenin is a key effector molecule in Wnt signaling pathway as T-cell factor (TCF) transcribes its target genes only when it binds to *β*-catenin [8, 12]. The mutations in adenomatous polyposis coli (APC) result in the stabilization and activation of *β*-catenin that leads to uncontrolled proliferation [13]. Therefore, overexpression of COX-2 and unrestrained Wnt signaling pathway play a crucial role in the pathogenesis of colorectal cancer.

Cancer is strongly influenced by various environmental factors, with diet being one of the major modifying agents [14]. Dietary fats play a crucial role both in the initiation and the inhibition of tumorigenesis [15]. Reports have shown that not only the amount but also the types of dietary fat differing in fatty acid composition are the important factors in pathogenesis of tumor. Experimental studies have observed that dietary supplementation of fish oil leads to the development of healthy state whereas corn oil augments the progression of diseases [16]. However, both of these are sources of essential polyunsaturated acids (PUFAs). Therefore, rather than their absolute intake, a balanced ratio of these oils in the diet is essential for normal growth and development [17]. The previous experiments conducted in our laboratory have demonstrated that the administration of fish oil (n-3 PUFA)/corn oil (n-6 PUFA) in 2.5/1 ratio has a better chemopreventive efficacy as compared to 1/1 in experimentally induced colorectal cancer by altering the cell cycle progression. Treatment with higher ratios of fish oil led to G1 arrest and a significant decrease in cell population in G2 M and S phase with reduced expression of cyclin D1 [18, 19]. Another study of our laboratory has shown that the differential chemopreventive action of fish oil and corn oil might be mediated through Ras induced Raf/Erk/Mek signaling pathway [20]. As the ceramide levels and nuclear localization of *β*-catenin play an essential role in the development of colon cancer, the present study was conducted to delineate the effect of both of these PUFAs on lipid mediated signaling and Wnt signaling. Hence, we analyzed the levels of ceramide, 15-deoxy PGJ_2_, COX-2 expression, alterations in Wnt signaling, and PPAR*γ* expression to outline the role of lipid mediated signalling events by fish oil in experimental model of colon carcinogenesis.

## 2. Materials and Methods

*N,N*′-Dimethylhydrazine dihydrochloride (DMH) was obtained from Sigma Chemical Company (St. Louis, USA). Monoclonal antibodies against COX-2 and horseradish peroxidase (HRP)-conjugated anti-mouse IgG were procured from Santa Cruz (CA, USA). Monoclonal antibody against PPAR*γ* was procured from Cayman Chemicals Company (Michigan, USA). Fluorescein isothiocyanate- (FITC-) conjugated goat anti-mouse IgG_1_ was bought from Bangalore Genei (Bangalore, India). Standards of ceramide and 15-deoxy PGJ_2_ were purchased from Sigma Chemical Company (St. Louis, USA). Maxepa fish oil [180 mg eicosapentaenoic acid (EPA) and 120 mg docosahexaenoic acid (DHA)/mL] was purchased from Merck Chemicals Limited (Goa, India) and corn oil [containing 58.8% linoleic acid, 26.4% oleic, 1.3% linolenic, and 12.8% saturated fatty acid] was obtained from Sigma Chemical Company (St. Louis, USA). The mineral mixture (Agrimin) was procured from Virbac Animal health India Pvt. Ltd. (Mumbai, India). All other chemicals used in the study were of analytical grade.

### 2.1. Animals and Diet

Male Wistar rats weighing 100–200 gm were obtained fromand housed in the Central Animal House, Panjab University, Chandigarh. The experimental protocols wereapproved by the “Institutional Animal Ethics Committee, Panjab University, Chandigarh” (1-12/IAEC 3/9/2009) and conducted according to guidelines of the “Indian National Science Academy” for the use and care of experimental animals. The animals were housed in polypropylene cages in the animal house and were acclimatized before being used in the experimental study. After one week of acclimatization, animals were randomly divided into the different groups and were fed experimental diet for four weeks. The composition of all experimental diets was adjusted so that animals in all the groups would consume the same amount of calories (Table 1) and has been described earlier [18].

### 2.2. Experimental Design

The male Wistar rats (*n* = 96) were divided into the six different experimental groups. The detailed experimental design was given by Sarotra et al., 2012 [18]. A brief summary is given below.

*Control Group.* These animals received purified diet and a weekly intraperitoneal injection of 1 mM ethylenediaminetetraacetic acid (EDTA with pH 7.4, a vehicle for DMH) for a period of 4 weeks. 

*DMH Treated.* The animals of this group received purified diet and a weekly intraperitoneal injection of DMH (20 mg/kg body weight) for a period of 4 weeks. 

*FO+CO(1 : 1)+EDTA.* A modified diet supplemented with 1 : 1 ratio of FO and CO was given to the animals along with a weekly intraperitoneal injection of EDTA for a period of 4 weeks. 

*FO+CO(1 : 1)+DMH.* Animals were given modified diet supplemented with 1 : 1 ratio of FO and CO and a weekly intraperitoneal injection of DMH for a period of 4 weeks. 

*FO+CO(2.5 : 1)+EDTA.* The animals of this group received modified diet supplemented with FO and CO in 2.5 : 1 ratio and a weekly intraperitoneal injection of EDTA for a period of 4 weeks.

*FO+CO(2.5 : 1)+DMH.* A modified diet supplemented with FO and CO in the ratio of 2.5 : 1 was given to the animals along with a weekly intraperitoneal injection of DMH for a period of 4 weeks. 

The animals which were sacrificed 48 h after the last EDTA/DMH injections constituted the initiation phase [21] and the animals kept for 12 weeks after the treatment regimen constituted the postinitiation phase study. All the animals were sacrificed by cervical dislocation.

### 2.3. Isolation of the Colonocytes

The colonocytes were isolated by the method of Sanders et al., 2004 [22]. The entire colon was cut longitudinally to expose lumen and placed in warm Ca^2+^ and Mg^2+^ free Hank's buffered salt solution (HBSS), 30 mmol/L EDTA, 5 mmol/L dithiothreitol (DTT), and 0.1% BSA (bovine serum albumin). After a 15 min shaking incubation at 37°C, the mucosal side was gently scraped to remove the intact crypts. The isolated cells were then centrifuged at 2200 rpm and washed twice in warm HBSS containing 1.3 mM CaCl_2_, 1 mM MgSO_4_, and 0.1% BSA. The cells were counted using a haemocytometer and their viability was checked by Trypan Blue Exclusion Method [23].

### 2.4. Lipid Profile

For the extraction of lipids, isolated colonocytes (1 – 2 × 10^6^ cells) were lysed with chloroform/methanol mixture. The samples were kept at room temperature for 30 min, centrifuged at 2000 rpm, and then filtered with Whatman filter paper number 1. The filtrate was pooled in the stoppered glass tubes, to which normal saline was added. The tubes were shaken vigorously and left overnight at 4°C to separate the aqueous and lipid layer. The upper aqueous layer was taken out using a glass pasteur pipette without disturbing the interfacial fluff. The samples were then dried under nitrogen gas and stored at −20°C.

### 2.5. Estimation of Phospholipids

Phospholipids were estimated by the method as described by McClare, 1971 [24]. The organic phosphorus of phospholipids is converted to inorganic phosphorus by digesting with perchloric acid. The inorganic phosphorus released was estimated by the method of Fiske and Subbarow, 1925 [25].

### 2.6. Estimation of Ceramide (Cer) by HPTLC

Cer was estimated by HPTLC using the modified method of Cordis et al., 1998 [26]. The lipid extracts were reconstituted in chloroform/methanol mixture and were fractionated by one-dimensional HPTLC techniques on silica gel 60 Å HPTLC plates (Whatman, Clifton, NJ). The plates were developed using toluene: methanol [8 : 2, v/v] mobile phase for Cer separation in a Camag HPTLC twin-trough chamber. The lipids were derivatized with primuline [100 mg primuline dissolved in 200 mL water and acetone (1 : 4, v/v)]. The plate was dipped (vertical speed: 2.5 cm/s and immersion time: 1 s) using the Chromatogram Immersion Device (CAMAG). The sample zones were quantified by linear scanning at 366 nm with a Camag TLC Scanner II, a mercury source, and slit dimension settings of length 4, width 4, and scanning rate of 20 mm s^−1^. The results of Cer were expressed as *μ*g of Cer/mg phospholipids.

### 2.7. Estimation of 15-Deoxy Prostaglandin J_2_ (15-deoxy PGJ_2_) by HPLC

The levels of 15-deoxy PGJ_2_ were estimated using HPLC by the method of Mazid et al., 2006 [27]. For the extraction of PGJ_2_, colon tissue was homogenized and sonicated in 0.1 M Tris (pH 7.4). Samples were then centrifuged at 800 g for 15 min. The supernatant was mixed with 1 M sodium acetate buffer (pH 3.5), and then the prostaglandins were extracted with ethyl acetate. The extracts were analyzed by RP-HPLC on the Waters HPLC system equipped with a 600 controller, 600 pump, and Waters 2996 photodiode array detector to which a L-column ODS was connected. The column was eluted with a mobile phase of acetonitrile/17 mM phosphoric acid (60 : 40, v/v) at a flow rate of 1 mL/min. The peak of 15d-PGJ_2_ was detectable by monitoring the absorbance at 230 nm. The data of 15-deoxy PGJ_2_ was expressed as ng of 15-deoxy PGJ_2_/gm tissue fraction.

### 2.8. Western Blotting of COX-2

Western blotting was carried out according to the procedure of Towbin et al., 1992 [28]. The tissue lysate was prepared in ice-cold lysis buffer (1% Triton X-100, 150 mM sodium chloride, 0.1% sodium dodecyl sulphate, 10 mM Tris pH 7.2, 50 mM sodium fluoride, 50 mM sucrose, and 20 *μ*M sodium orthovanadate). The lysate was incubated on ice for 60 min and was centrifuged at 12,000 g for 20 min. The supernatant was collected and the protein content was estimated according to Lowry et al., 1951 [29]. 100 *μ*g of protein was loaded onto the gel and was run along with prestained protein marker. The protein from the gel was transferred onto the PVDF membrane. The membrane was blocked in 3% nonfat dry milk in TBS for 1 hour at room temperature and was then incubated overnight at 4°C with monoclonal antibody against COX-2 which was diluted (1 : 100) in the blocking buffer. The membrane was then washed three times in TBST and then incubated with the corresponding HRP conjugated secondary antibody diluted (1 : 250) in blocking buffer for 2 hours at room temperature with constant agitation. The membrane was then again washed in TBST. The protein was visualized with PBS containing DAB (1 mg/mL) and H_2_O_2_, substrate for HRP. The reaction was stopped by washing the membrane with PBS after which the membrane was photographed.

### 2.9. Immunohistochemical Analysis of *β*-Catenin

2-3 *μ*m thick sections were mounted on poly-L-lysine coated slides. The slides were heated at 65°C before deparaffinization in xylene. The slides were rehydrated with serial alcohol solutions (100%, 90%, 70%, 50%, and 30%). Endogenous peroxidase activity was quenched by incubating the slides with 3% H_2_O_2_ (in methanol) for 20 min at 4°C. The sections were blocked using 2% BSA in PBS for 30 min at room temperature. Antigen retrieval was done with retrieval buffer (pH 6.0) by using the microwave for 5 min. The slides were allowed to cool for 20 minutes. Following antigen retrieval, the sections were incubated with the diluted antibody *β*-catenin (1 : 100) for overnight at 4°C in a humid chamber. The slides were washed in PBS and followed by incubation for 2 hours with HRP-conjugated anti-rabbit antibody (1 : 100) for *β*-catenin at 37°C in a humid chamber. The slides were visualized using DAB and H_2_O_2_. Sections were then counterstained with haematoxylin for 2 min, followed by rinsing in deionized H_2_O. Slides were dehydrated and mounted with DPX for analysis. Images were acquired and analyzed using the Nikon Eclipse 80*i* microscope (Japan) and Northern Eclipse Imaging Elements-D (NIS-D) software.

### 2.10. Analysis of PPAR*γ* by Flow Cytometer

The isolated colonocytes were fixed in 4% paraformaldehyde for 20 minutes at room temperature. After washing with PBS twice, the colonocytes were permeabilized with 100% ice-cold methanol (added drop wise) and left for 15 min at −20°C. The cells were washed again in cold PBS twice. Approximately 1 × 10^6^ cells were added to a FACS tube, resuspended in saponin buffer (PBS containing 0.1% saponin and 2% BSA), and incubated for 30 min at 4°C. The colonocytes were then incubated with diluted PPAR*γ* (1 : 100) monoclonal antibody for 30 min at room temperature and then washed with saponin buffer. The colonocytes were then incubated with diluted FITC conjugated secondary antibody for 45 min at room temperature. The cells were washed once with saponin buffer and then with PBS. The cells were resuspended in PBS. The acquisition from each sample was conducted on FACS Canto (BD Biosciences, USA) and the collected data was analyzed using the BD FACS Diva software. The results of PPAR*γ* were represented as the mean of Net MFI (MFI of cells treated with Ab- MFI of cells only).

### 2.11. Statistical Analysis

The results were expressed as Mean ± S.D. The differences between the groups were assessed by ANOVA after ascertaining normality by Q-Q plot. The statistical significance was determined by one-way ANOVA with Bonferroni's multiple comparison post hoc tests, and differences were considered significant for *P* < 0.05.

## 3. Results

### 3.1. Effect of Different Ratios of Fish Oil and Corn Oil on Ceramide Levels

**Figure 1:**
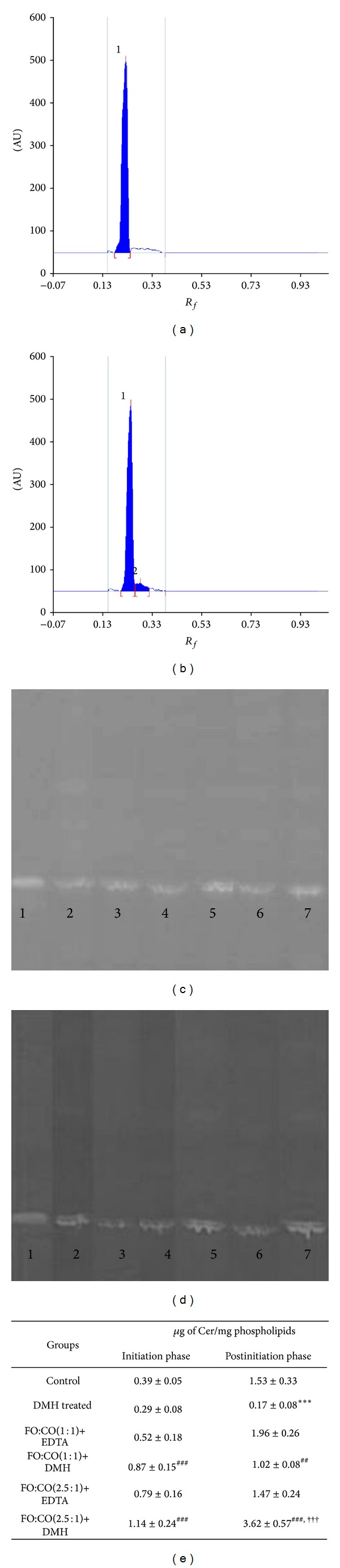
Effect of different ratios of fish oil and corn oil on ceramide levels in experimentally induced colon cancer. Chromatogram depicts (a) peak of ceramide standard, (b) peak of test sample, (c) band intensity in different groups of initiation phase, and (d) band intensity in different groups of postinitiation phase. (e) represents expression of ceramide in the initiation and postinitiation phase. Lane 1-band intensity of std. ceramide, 2-control, 3-DMH treated, 4-FO+CO(1 : 1)+EDTA, 5-FO+CO(1 : 1)+DMH, 6-FO+CO(2.5 : 1)+EDTA, and 7-FO+CO(2.5 : 1)+DMH. The results are expressed as Mean ± S.D. for *n* = 8. ***P < 0.001 with respect to control, ^###^ *P* < 0.001 with respect to DMH, ^##^ *P* < 0.01 with respect to DMH, and ^†††^ *P* < 0.001 with respect to FO : CO(1 : 1)+DMH.

As ceramide, a bioactive lipid, has been proposed to play important role in growth arrest, differentiation, and apoptosis, we thought whether the different ratios of fish oil and corn oil might alter the ceramide levels in the initiation and postinitiation phase of rodent model of colon cancer. The levels of ceramide were measured by HPTLC and peak of ceramide is shown in Figures 1(a) and 1(b). The ceramide levels of DMH treated animals of both initiation and postinitiation phases were compared with the corresponding control animals. It has been shown that, on treatment with DMH in the initiation phase, there was no significant alteration in the levels of ceramide in comparison to control animals; however, it was decreased markedly in the postinitiation phase. On receiving FO+CO(1 : 1)+DMH and FO+CO(2.5 : 1)+DMH in both phases, ceramide levels were augmented significantly with respect to DMH treated animals. The elevation in the levels of ceramide was more marked with FO+CO(2.5 : 1)+DMH as compared to FO+CO(1 : 1)+DMH. These observations clearly suggest that the supplementation of dietary fish oil modulates the lipid mediator, ceramide, in DMH induced experimental colon carcinogenesis.

### 3.2. Effect of Different Ratios of Fish Oil and Corn Oil on the Expression of COX-2

**Figure 2:**
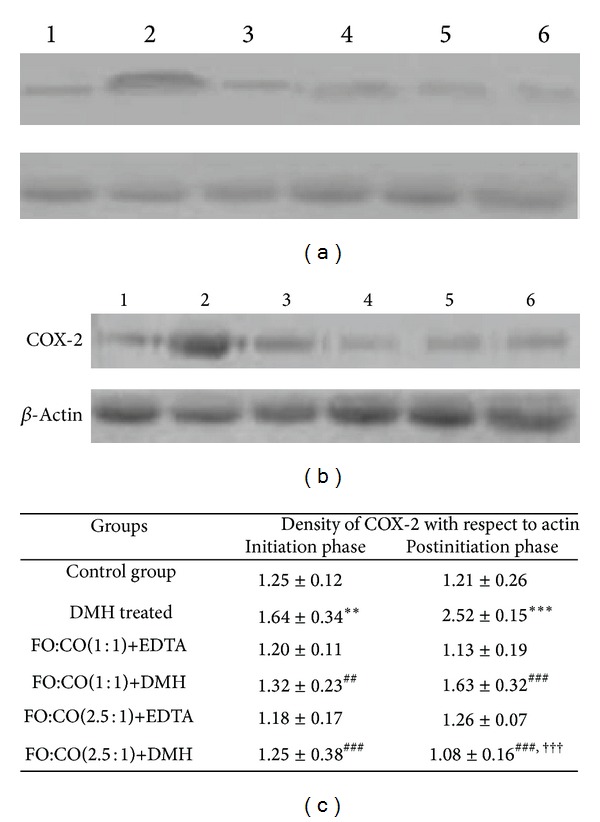
Alterations on the expression of COX-2 on administration of both of the ratios of fish oil and corn oil in experimentally induced colon cancer. (a) depicts band intensity in the initiation phase of different groups and (b) represents band intensity in postinitiation phase. (c) depicts quantitative analysis of COX-2 expression in the initiation and postinitiation phase. Lane 1-band intensity of control, 2-DMH treated, 3-FO+CO(1 : 1)+EDTA, 4-FO+CO(1 : 1)+DMH, 5-FO+CO(2.5 : 1)+EDTA, and 6-FO+CO(2.5 : 1)+DMH. The results are expressed as Mean ± S.D. for *n* = 4. ***P < 0.001 with respect to control, **P < 0.01 with respect to control, ^###^ *P* < 0.001 with respect to DMH, ^##^ *P* < 0.01 with respect to DMH, and ^†††^ *P* < 0.001 with respect to FO : CO(1 : 1) + DMH.

As the overexpression of another lipid mediator, COX-2, has been considered to be the hallmark of colorectal cancer progression, next we examined the expression of COX-2 enzyme. The expression of COX-2 is represented in Figure 2. In the present study, COX-2 enzyme was increased significantly on treatment with DMH as compared to control animals in both of the phases. On receiving the FO+CO(1 : 1)+DMH and FO+CO(2.5 : 1)+DMH, the expression of COX-2 was decreased in both phases as compared to DMH treated animals. The effect was more marked with FO+CO(2.5 : 1)+DMH in the postinitiation phase.

### 3.3. Effect of Different Ratios of Fish Oil and Corn Oil on the Expression of 15-Deoxy PGJ_2_

**Figure 3:**
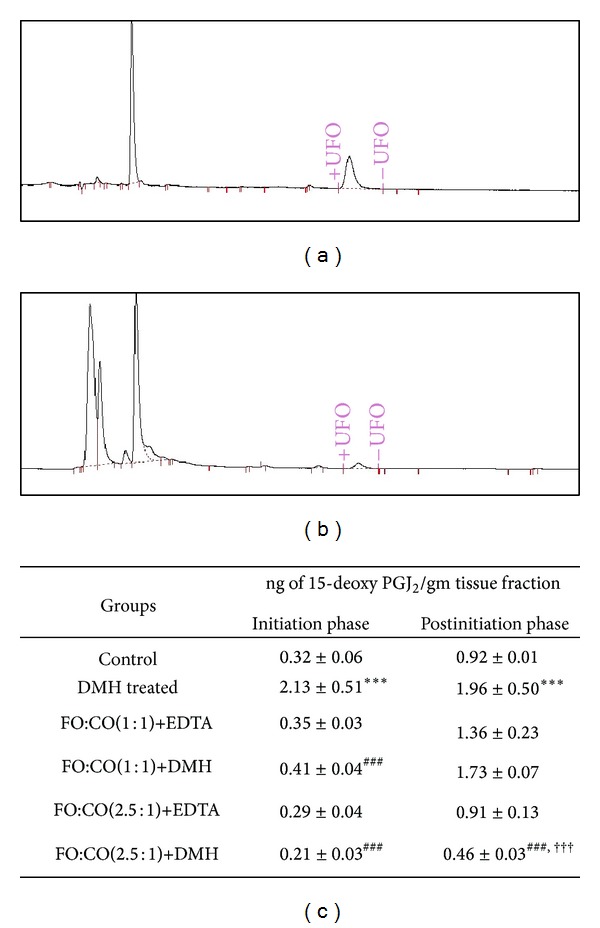
Downregulation of 15-deoxy PGJ_2_ on administration of different ratios of fish oil and corn oil in experimentally induced colon cancer. Chromatogram represents (a) peak of standard 15-deoxy PGJ_2_ and (b) peak of test sample. (c) Table depicting the levels of 15-deoxy PGJ_2_ in both the initiation and postinitiation phase. The results are expressed as Mean ± S.D. for *n* = 8. ***P < 0.001 with respect to control, ^###^ *P* < 0.001 with respect to DMH, and ^†††^ *P* < 0.001 with respect to FO : CO(1 : 1) + DMH.

Since the overexpression of COX-2 leads to the formation of proinflammatory eicosanoids and it has been observed in the present study that both ratios reduced the expression of COX-2, we further examined whether these fatty acids in both ratios alter the expression of 15-deoxy PGJ_2_. The levels of 15-deoxy PGJ_2_ were analyzed by HPLC and the representative peak of standard and sample is given in Figures 3(a) and 3(b). On treatment with DMH, the levels of 15-deoxy PGJ_2_ were elevated considerably as compared to control animals in both phases. However, on receiving both of the ratios of fish oil and corn oil in initiation and postinitiation phase, the levels of 15-deoxy PGJ_2_ were decreased significantly with respect to DMH treated animals. The decrease was more pronounced with FO+CO(2.5 : 1)+DMH.

### 3.4. Effect of Different Ratios of Fish Oil and Corn Oil on the Levels of PPAR*γ*

Since 15-deoxy PGJ_2_ is the natural ligand of PPAR*γ*, we next determined the effect of these PUFAs on PPAR*γ* in experimentally induced colon cancer. It has been demonstrated that, in the initiation phase, there was no significant alteration in the expression of PPAR*γ* on treatment with DMH in comparison to control animals; however, in the postinitiation phase, expression of PPAR*γ* was considerably augmented (Table 2). On receiving FO+CO(1 : 1)+DMH and FO+CO(2.5 : 1)+DMH in the initiation phase, the expression of PPAR*γ* was elevated significantly with respect to DMH treated animals, whereas it was decreased significantly in the postinitiation phase.

### 3.5. Effect of Different Ratios of Fish Oil and Corn Oil on the Expression of *β*-Catenin

**Figure 4:**
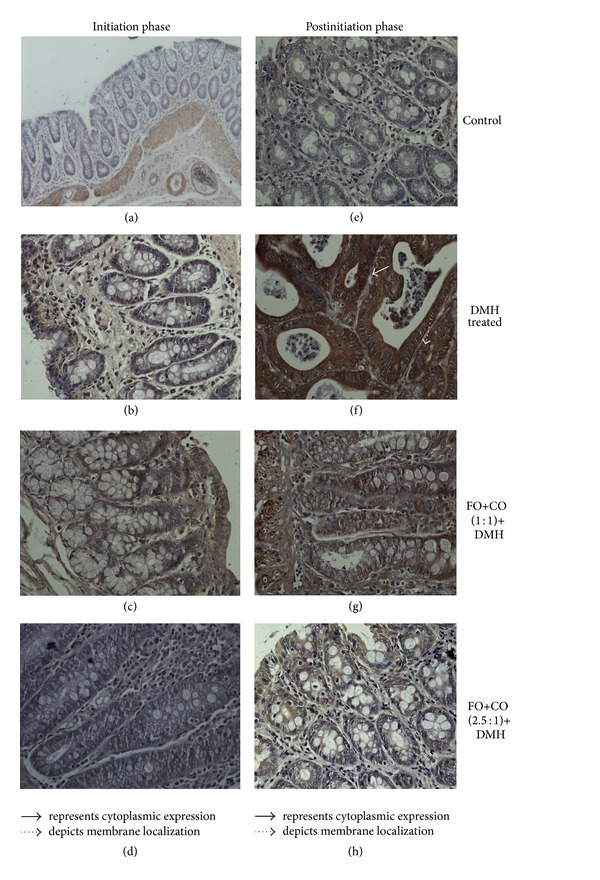
Effect of different ratios of fish oil and corn oil on the expression and nuclear localization of *β*-catenin in initiation and postinitiation phase. ⟶ represents cytoplasmic expression and ⇢ depicts membrane localization.

In order to further corroborate the effects of dietary fish oil and corn oil, we evaluated the nuclear localization of *β*-catenin. Overexpression of prostaglandins induces the stabilization of *β*-catenin, which interacts with Tcf/Lef1 transcription factors and activates the expression of downstream genes. The results on the expression and localisation of *β*-catenin in colon tissue are summarized in Table 3 and Figure 4. As shown by the immunohistochemical staining, *β*-catenin was localized at the membrane of epithelial cells in colonic mucosa of control animals. On treatment with DMH, an increase in the expression as well as nuclear localization of *β*-catenin was observed (Figures 4(b) and 4(f)) in both phases as compared to control animals. On treatment with FO+CO(1 : 1)+DMH, expression of *β*-catenin was reduced in both membrane and cytoplasm as compared to DMH treated animals. In contrast, animals receiving FO+CO(2.5 : 1)+DMH have demonstrated moderate immunoreactivity of *β*-catenin on the membrane only. The nuclear translocation of *β*-catenin was found to be inhibited on treatment with both of the ratios in both phases as compared to DMH treated animals.

## 4. Discussion

The sphingolipid ceramide is an important molecule that regulates diverse signaling pathways by triggering specific protein targets such as phosphatases and kinases. Activation of phosphoprotein phosphatase 1 (PP1) by ceramide induces dephosphorylation of retinoblastoma (Rb), thus implicating it in cell cycle arrest. Ceramide also induces the apoptosis which may be mediated through SAPK/JNK signaling pathway [30]. Here we reported that treatment with DMH resulted in a decrease in ceramide in the initiation phase but the decrease was not significant with respect to control animals. However, there was a significant decrease in ceramide levels in the postinitiation phase as compared to control animals. Ceramide leads to a decline in the proliferative potential and also affects the cell differentiation [31, 32]. Hence, the reduced level of ceramide in the present study suggests the progression of colorectal cancer in the current model. The diminished levels of ceramide in the present study are in corroboration with the results of our previous study which had shown the increased cell proliferation in a similar model [20]. On treatment with both ratios of fish oil and corn oil, the levels of ceramide were increased in both phases in a dose dependent manner which should lead to the activation of apoptosis or inhibition of cell proliferation which indeed was the observation in our previous study [20]. It has been reported earlier also that supplementation of DHA or EPA leads to an increased ceramide formation in breast cancer cells [33]. Elevated levels of ceramide are considered to be highly effective in inducing apoptosis and preventing colon cancer [32].

The reports have demonstrated that deprivation of DHA and EPA (n-3PUFAs) activates arachidonic acid metabolizing enzymes like COX-2 which lead to the development of cancer [34]. The data of the current study has demonstrated that, on treatment with DMH, the levels of COX-2 were increased in both the initiation and the postinitiation phase in comparison to control animals. An elevation in COX-2 activity results in cell proliferation, increases the metastatic potential, prevents the apoptosis, and, therefore, has a significant role in colon carcinogenesis [35, 36]. According to the canonical process of signal transduction, the observed increase in expression of COX-2 upon treatment with DMH is expected to be correlated with enhanced downstream signaling. The activated COX-2 would lead to an increase in the production of prostaglandins, for example, 15-deoxy PGJ_2_, observed in the current study. We observed an increase in 15-deoxy PGJ_2_ levels on treatment with DMH in both the initiation and the postinitiation phase as compared to control animals. It has been reported that inflammatory prostanoids activate G protein-coupled receptor and lead to the association of *α* subunit of G protein with the signaling domain of axin; this results in the release of GSK-3*β* from its complex, hence leading to the accumulation and nuclear localization of *β*-catenin [8, 10]. We have also found the enhanced expression of *β*-catenin in membrane, cytoplasm, and nucleus on treatment with DMH in both phases. The elevation in the expression of *β*-catenin was more pronounced in the postinitiation phase. The nuclear translocation of *β*-catenin induces the target gene expression involved in cellular proliferation, such as c-myc, cyclin D1, and PPAR*γ* [37–39]. The previous study conducted in our laboratory has also reported an increase in the levels of cyclin D1 levels on treatment with DMH and the increase was more significant in the postinitiation phase [19]. Hence, the enhanced activity of COX-2, 15-deoxy PGJ_2_, and aberrant Wnt signaling in the current study suggests the involvement of this crucial signaling pathway in DMH induced colon cancer.

On treatment with fish oil and corn oil, the expression of COX-2 and 15-deoxy PGJ_2_ was decreased in both phases as compared to DMH treated animals. The decrease was more significant with FO+CO(2.5 : 1)+DMH as compared to FO+CO(1 : 1)+DMH. It has been observed that, on treatment with FO+CO(1 : 1)+DMH, *β*-catenin was expressed in membrane and cytoplasm; however, treatment with FO+CO(2.5 : 1)+DMH and *β*-catenin was found to be expressed on membrane only in both phases. Hence, the localization of *β*-catenin from cytoplasm to nucleus was inhibited, suggesting the inactivation of Wnt signaling in a dose dependent manner. It has been observed earlier also that supplementation of DHA inhibits COX-2 expression and induces apoptosis in WM266-4 metastatic melanoma cell line [40]. DHA supplementation also induced the formation of *β*-catenin/axin/GSK-3*β* complex and, hence, inhibited nuclear localization of *β*-cateninin hepatocellular carcinoma cell line [41]. Furthermore, it has been reported that n-3 PUFAs decreased PGE_2_ signaling through downregulation of COX-2 in HCC cell line [41]. This is the first study in the animal model which shows that the supplementation of fish oil and corn oil in different ratios exerts the differential effect in the inhibition of COX-2 expression and Wnt signaling in a dose and time dependent manner.

As PPAR*γ* is the direct receptor of inflammatory prostanoids, that is, 15-deoxy PGJ_2_; therefore, the expression of PPAR*γ* was also analyzed. Here, the results have shown that, on treatment with DMH, the expression of PPAR*γ* was slightly decreased in initiation phase as compared to control animals whereas it was increased in the postinitiation phase. A marginal decrease in the expression of PPAR*γ* in the initiation phase might be related to the early stages of colon cancer. The results of an elevated PPAR*γ* in postinitiation phase in the current study are contrary to other reports which have revealed a decreased expression of PPAR*γ* in cancer [42, 43]. However, in the presence of mutation in APC gene, PPAR*γ* has been reported to be elevated in the later stages and promote the progression of colon cancer [44, 45]. It has been documented earlier also that PPAR*γ* can act as a tumour promoter, only in the presence of APC mutation or aberrant Wnt signaling pathway which indeed is the observation in the current study [46]. Activation of PPAR*γ* during tumour progression is deleterious as it also has an important role in the modulation of tumour microenvironment by altering the immune status in epithelial cancers [47]. Another reason for the activation of PPAR*γ* in the present study might also be related to the elevated levels of its ligand 15-deoxy PGJ_2_ by COX-2.

The results of PPAR*γ* in the present study suggest that, on treatment with FO+CO(1 : 1)+DMH and FO+CO(2.5 : 1)+DMH, PPAR*γ* was induced in comparison to DMH treated animals in the initiation phase. Even though there is a decrease in the expression of COX-2, 15-deoxy PGJ_2_, and nuclear localization of *β*-catenin, we still observed an elevation in the expression of PPAR*γ*. This could be related to the direct activation of PPAR*γ* by DHA and its metabolites 17-OH DHA as reported earlier [48]. On treatment with FO+CO(1 : 1)+DMH and FO+CO(2.5 : 1)+DMH in the postinitiation phase, the levels of PPAR*γ* were found to be decreased as compared to DMH treated animals. The decrease in PPAR*γ* was more pronounced with FO+CO(2.5 : 1)+DMH as compared to FO+CO(1 : 1)+DMH. The reduced expression of PPAR*γ* in the current study might be related to the decrease in expression of COX-2, 15-deoxy PGJ_2_, and nuclear localization of *β*-catenin. As the supplementation of fish oil and corn oil reverses the expression of PPAR*γ* as compared to DMH treated animals, it reflects the role of PPAR*γ* in the chemoprevention offered by the ratios of these PUFAs in colon carcinogenesis.

## 5. Conclusion

In summary the results of the current study suggest that, on supplementation of fish oil, ceramide levels were increased whereas expression of COX-2, 15-deoxy PGJ_2_, and Wnt/*β*-catenin signaling was reduced in both phases with the effect being more pronounced with the higher ratio, that is, FO+CO(2.5 : 1)+DMH in the postinitiation phase. Hence, it can be concluded that fish oil exerts dose and time dependent chemopreventive effect in experimentally induced colon cancer which may be mediated through an inhibition in lipid mediated signaling and aberrant Wnt signaling.

## Figures and Tables

**Table 1:** Composition of the diet.

Synthetic diet content (%)	Control	DMH treated	FO + CO (1 : 1) + EDTA	FO + CO (1 : 1) + DMH	FO + CO + (2.5 : 1) + EDTA	FO + CO (2.5 : 1) + DMH
Casein	20	20	23.5	23.5	23.5	23.5
Fat	5 (soya oil)	5 (soya oil)	5 + 7.5 CO + 7.5 FO	5 + 7.5 CO + 7.5 FO	5 + 4.3 CO + 10.7 FO	5 + 4.3 CO + 10.7 FO
Carbohydrate	65	65	44.72	44.72	44.72	44.72
Fibre	5	5	5.9	5.9	5.9	5.9
Mineral mix.	3.8	3.8	4.46	4.46	4.46	4.46
Vitamin mix.	1.0	1.0	1.18	1.18	1.18	1.18
Choline chloride	0.2	0.2	0.24	0.24	0.24	0.24

**Table 2:** Effect of fish oil and corn oil on PPAR*γ* expression in experimentally induced colon cancer.

Groups	Net MFI of PPAR*γ*	
	Initiation phase	Postinitiation phase
Control group	129.33 ± 7.11	148.50 ± 11.22
DMH treated	116.33 ± 7.89	356.83 ± 29.48***
FO : CO (1 : 1) + EDTA	276.83 ± 18.47	153.89 ± 9.32
FO : CO (1 : 1) + DMH	239.60 ± 34.01^###^	207.33 ± 13.38^###^
FO : CO (2.5 : 1) + EDTA	162.60 ± 8.73	163.00 ± 16.31
FO : CO (2.5 : 1) + DMH	156.33 ± 16.42^###,†††^	134.67 ± 8.09^###,†††^

The results are expressed as Mean ± S.D. for *n* = 8.

***P < 0.001 with respect to control, ^###^ *P* < 0.001 with respect to DMH, and ^†††^ *P* < 0.001 with respect to FO : CO (1 : 1) + DMH.

**Table 3:** Effect of fish oil and corn oil on expression and nuclear localization of *β*-catenin in the colon cancer.

Groups	Initiation phase (% of *β*-catenin positive cells)			Postinitiation phase (% of *β*-catenin positive cells)		
	Membranous	Cytoplasmic	Nuclear	Membranous	Cytoplasmic	Nuclear
Control group	5.85 ± 3.95	—	—	8.56 ± 2.35	—	—
DMH treated	12.47 ± 1.29***	18.36 ± 5.34***	16.89 ± 1.56***	45.32 ± 4.22***	19.03 ± 2.94***	15.45 ± 2.34***
FO : CO (1 : 1) + EDTA	6.71 ± 2.54	—	—	10.43 ± 0.88	—	—
FO : CO (1 : 1) + DMH	8.56 ± 1.58^###^	6.67 ± 2.69^###^	—	20.68 ± 2.36^###^	5.96 ± 3.87^###^	3.40 ± 0.56^###^
FO : CO (2.5 : 1) + EDTA	8.36 ± 3.62	—	—	7.68 ± 1.65	—	—
FO : CO (2.5 : 1) + DMH	11.91 ± 2.56^†^	—	—	4.56 ± 1.23^###^	—	—

The results are expressed as Mean ± S.D. for *n* = 8.

***P < 0.001 with respect to control, ^###^ *P* < 0.001 with respect to DMH, and ^†^ *P* < 0.05 with respect to FO : CO (1 : 1) + DMH.
